# New records and description of two new species of carideans shrimps from Bahía Santa María-La Reforma lagoon, Gulf of California, Mexico (Crustacea, Caridea, Alpheidae and Processidae)

**DOI:** 10.3897/zookeys.671.9081

**Published:** 2017-04-27

**Authors:** José Salgado-Barragán, Manuel Ayón-Parente, Pilar Zamora-Tavares

**Affiliations:** 1 Laboratorio de Invertebrados Bentónicos, Unidad Académica Mazatlán, Instituto de Ciencias del Mar y Limnología, Universidad Nacional Autónoma de México, México; 2 Centro Universitario de Ciencias Agropecuarias, Universidad de Guadalajara, México

**Keywords:** Caridea, Crustacea, genetic analysis, Mexican Pacific, new species

## Abstract

Two new species of the family Alpheidae: *Alpheus margaritae* **sp. n.** and *Leptalpheus melendezensis* **sp. n.** are described from Santa María-La Reforma, coastal lagoon, SE Gulf of California. *Alpheus margaritae* **sp. n.** is closely related to *A. antepaenultimus* and *A. mazatlanicus* from the Eastern Pacific and to *A. chacei* from the Western Atlantic, but can be differentiated from these by a combination of characters, especially the morphology of the scaphocerite and the first pereopods. *Leptalpheus melendezensis* **sp. n.** resembles *L. mexicanus* but can be easily differentiated because *L. melendezensis* **sp. n.** has the anterior margin of the carapace broadly rounded and has only one spine on the mesial margin of ischium in the major cheliped, versus an acute rostrum and an unarmed major cheliped. Additionally, a phylogenetic analysis was used to explore the relationships of these two new taxa. These results show that *Alpheus margaritae* **sp. n.** and *Leptalpheus melendezensis* **sp. n.** are indeed related to the species against which we are comparing them, and demonstrate that they can be considered as different species. Additional specimens of Leptalpheus cf. mexicanus, *Ambidexter panamensis* and *A. swifti* are recorded for the first time in the Santa María-La Reforma coastal lagoon.

## Introduction

Caridea is the second most species-rich taxon amongst decapod crustaceans (De Grave and Fransen 2011) including approximately 3450 marine, brackish and freshwater living species. In the Eastern Tropical Pacific (ETP) this group is very well studied and it is the most diversified group of shrimps with a total of 204 species recorded in 2003 (Wicksten and Hendrickx 2003). Since then, at least 26 new species have been described in this region belonging to the genera *Alpheus* Fabricius, 1798 (Anker et al. 2007a; 2007b; 2009; Anker and Pachelle 2013; 2015; Bracken-Grissom and Felder 2014), *Bathystylodactylus* Hanamura & Takeda, 1996 (Wicksten and Martin 2004), *Glyphocrangon* A. Milne-Edwards, 1881 (Hendrickx 2010), *Leptalpheus* Williams, 1965 (Anker 2011; Salgado-Barragán et al. 2014; Anker and Lazarus 2015a), *Leslibetaeus* Anker, Poddoubtchenko & Wehrtmann, 2006 (Anker et al. 2006), *Lysmata* Risso, 1816 (Anker et al. 2009), *Ogyrides* Stebbing, 1914 (Ayón-Parente and Salgado-Barragán 2013), *Pontonia* Latreille, 1829 (Marin and Anker 2008), *Prionocrangon* Wood-Mason,1891 *in* Wood-Mason and Alcock 1891 (Hendrickx and Ayón-Parente 2012), *Salmoneus* Holthuis, 1955 (Anker and Lazarus 2015b), *Synalpheus* Spence Bate, 1888 (Hermoso and Alvarez 2005; Hermoso-Salazar and Hendrickx 2006), *Triacanthoneus* Anker, 2010 (Anker 2010) and *Typton* Costa, 1844 (Ayón-Parente et al. 2015). Despite the knowledge of caridean species recorded in the ETP, there remains a lack of knowledge about the biogeography, distribution and ecology of the group (Hendrickx and Wicksten 2011), particularly regarding infaunal and lagoon species.

The phylogenetic relationships of Alpheidae have been previously assessed in several studies (Knowlton et al. 1998; Williams et al. 2001; Mathews et al. 2002; Anker 2012; Anker et al. 2007; 2008a; 2008b; 2008c; Mathews and Anker 2009; Bracken-Grissom and Felder 2014; Bracken-Grissom et al. 2014). These studies have focused on the resolution of the relationships between species complexes within *Alpheus*. With regards to *Leptalpheus* species, there are no previous phylogenetic analyses. The application of molecular analyses has changed the way of describing and cataloging biological diversity. The use of these approaches has led to new inferences in the evolutionary dynamics of lineages and the role of morphological and genetic evolution (Mathews and Anker 2009).

The Santa María-La Reforma coastal lagoon is one of the largest coastal systems along the NW coast of Mexico. This coastal complex has been identified as one of the richest and most productive in the region, with great considerable importance for the shrimp and fish captures in the Gulf of California (Amezcua et al. 2006; Ruiz-Luna et al. 2010) and also as a support system for seabird populations, as it includes the largest breeding site of several species in Western Mexico (Castillo-Guerrero et al. 2014). Notwithstanding its importance, information about the carcinological fauna of this lagoon is limited and restricted to commercially harvested species (*Callinectes* spp. and penaeid shrimps) (Ruiz-Luna et al. 2010; Rodríguez-Domínguez et al. 2012).

A number of specimens of caridean shrimps were collected during surveys of infaunal invertebrates at several locations of the Bahía Santa María-La Reforma lagoon. After a detailed review of the specimens, we determined that they belong to five species of the genus *Alpheus*, *Leptalpheus* and *Ambidexter* Manning & Chace, 1971. The morphological analysis of the organisms and DNA sequences obtained from two regions: 16S and COI helped us to confirm that two species are undescribed species, which are herein described and illustrated. *Alpheus margaritae* sp. n. is related to the *A. antepaenultimus* species complex in the Eastern Pacific and to *A. chacei* in the western Atlantic. On the other hand, *Leptalpheus melendezensis* sp. n. seems to be related to *L. forceps* and *L. mexicanus*. The other species comprise new distribution records in the Mexican Pacific.

## Materials and methods

From June 2013 to March 2015, a series of surveys was carried out in Bahía Santa María-La Reforma lagoon (SE Gulf of California), in order to collect infaunal crustaceans. Collecting sites were characterized by mud, sand and sandy-mud bottoms with profuse burrow openings of mud shrimps identified as *Neotrypaea tabogensis* (Sakai, 2005), *Neotrypaea* sp., and Callichirus cf. major Stimpson, 1866. The organisms were collected using a yabby type suction pump operated at intertidal level (0–40 cm depth). Specimens were preserved in 75% alcohol and deposited in the Regional Collection of Marine Invertebrates, Instituto de Ciencias del Mar y Limnología, Universidad Nacional Autónoma de México, in Mazatlán, Sinaloa, Mexico (**EMU**). One male and one female paratypes of *A. margaritae* sp. n. were deposited in the Colección Nacional de Crustáceos, Instituto de Biología, Universidad Nacional Autónoma de México, Ciudad de México, Mexico (**CNCR**). Carapace length of all organisms (**CL**), in mm, was measured from the apex of the rostrum to the median posterior margin of carapace.

Total genomic DNA of alpheids from Bahía Santa María-La Reforma and from the Regional Collection of Marine Invertebrates was extracted from muscle tissue of the walking legs. DNA sequences were obtained from two regions, 16S and COI. The Internal Transcribed Spacer (ITS) sequences were used to estimate the phylogenetic relationships of *L. melendezensis* sp. n. and L. cf. mexicanus Ríos and Carvacho, 1983 and COI for *A. margaritae* sp. n. The ITS was amplified with the primers 16SH2 (5'-AGATAGAAACCAACCTGG-3') and 16Sar (5'-CGCCTGTTTATCAAAAACAT-3'), and the following thermal cycler conditions 10 min at 94 °C; annealing for 38–42 cycles: 1 min at 94 °C, 1 min at 45–48 °C, 2 min at 72 °C; final extension of 10 min at 72 °C. For the COI region we used the universal primers LCO1490 and HCOI 2198 (Folmer et al. 1994). The PCR took place in a final volume of 20 µL consisting of 12.1 µL of H_2_0, 2 µL of reaction buffer, 0.8 µL of dNTPs, 0.5 µL of each primer, 0.5 µL of MgCl_2_, 0.3 µL Platinum Taq, and 2 µL total DNA. The thermal cycler program was 95 °C for 2 minutes; followed by six cycles at 95 °C for 15 seconds, 45 °C for 15 seconds, 72 °C for 1 minute, then 30 cycles at 95 °C for 15 seconds, 48 °C for 15 seconds, 72 °C for 1 minute, and a final extension step of 72 °C for 3 minutes. The out-group *Alpheopsis trigona* (Rathbun, 1901) and some sequences of the in-group, for the two regions, were obtained from GenBank (Table 1). Amplification products were purified with the enzyme Exosap IT at a volume of 1 µL per 10 µL amplified. These samples were sequenced with the TaqBigDye Terminator Cycle Sequencing kit (Perkin Elmer Applied Biosystems, Foster City, USA), purified with Illustra Autoseq G50 columns and visualized on a 310ABI DNA Sequencer (Perkin Elmer Applied Biosystems, Foster City, USA). The DNA sequences were edited with Chromas Pro 1.41 (McCarthy 1996-1998). Matrices with the DNA sequences for each of the regions were generated using PhyDE 0.9971 (Müller et al., 2011). DNA sequences were aligned using SEAVIEW 4 (Gouy and Gascuel, 2010) with the MUSCLE algorithm (Edgar 2004). Aligned sequences were analyzed using jModelTest 0.1.1 (Posada 2008) to obtain the best-fit nucleotide substitution models to the data, based on the Akaike Information Criterion (Posada and Crandall 1998). Molecular data were analyzed using maximum likelihood (ML) in RAxML 7.0.0 (Stamatakis et al. 2006) using the CIPRES platform, which has the advantage of reducing the execution time of the analysis (Miller et al. 2010). The ML tree was obtained and to assess nodal support a resampling of 1,000 rapid bootstrap inferences was done.

**Table 1.** Sequence data of *Alpheus* and *Leptalpheus* species, voucher number, and Genbank accession.

	16S		COI				
*Alpheopsis trigonus*	EU868633^+^		AF309946*				
*Alpheus antepaenultimus*			AF309873*	AF309874*	AF309875*	AF309876*	AF308989*
*Alpheus chacei*	JX286606^¥^		AF309884*				
*Alpheus cylindricus*			AY344733*	AF309882*	AF309891*		
*Alpheus colombiensis*			AF309886*	AF309885*	AF308981*		
*Alpheus estuariensis*			AF309894*	AF309895*	AF309896*		
*Alpheus floridanus*			KP100633^§^	KP100634^§^	KP100635^§^		
*Alpheus hephaestus*			KP100629^§^	KP100630^§^	KP100631^§^		
*Alpheus latus*			AF309909*				
*Alpheus lottini*			AF309910*	KJ155615	KJ155539		
*Alpheus panamensis*			AF309923*				
*Alpheus margaritae* sp. n.	KY780077^£^	KY780078^£^	KY780079^£^	KY780080^£^			
*Alpheus saxidomus*			AF309934*	AF309935*			
*Leptalpheus axianassae*	EU868764^+^						
*Leptalpheus corderoae*	KY674509^£^						
*Leptalpheus forceps*	EU868763^+^						
*Leptalpheus hendrickxi*	KY674510^£^						
*Leptalpheus melendezensis* sp. n.	KY674512^£^		KY780076^£^				
*Leptalpheus mexicanus*	KY674511^£^						

Sequences authors: ^*^ Williams et al., 2001;

^+^ Bracken et al., 2009;

^¥^ Almeida et al., 2013;

^§^ Bracken-Grissom et al., 2014;

^£^ this study.

## Systematic account

### Family Alpheidae Rafinesque, 1815

#### Genus *Alpheus* Fabricius, 1798

##### Alpheus margaritae sp. n.

Taxon classificationAnimaliaDecapodaAlpheidae

http://zoobank.org/6956A091-E7C7-4313-975F-5DC82A4CC409

**Figure 1.**
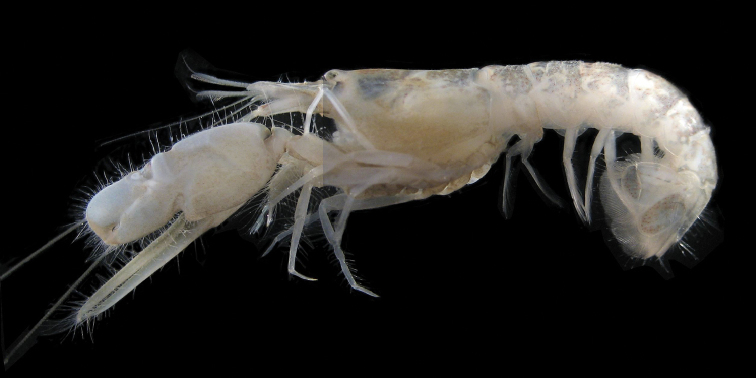
*Alpheus margaritae* sp. n. Female paratype from Isla Costa Azul, Santa María-La Reforma costal lagoon, Sinaloa, Mexico (EMU-10581), lateral view, color in life.

**Figure 2.**
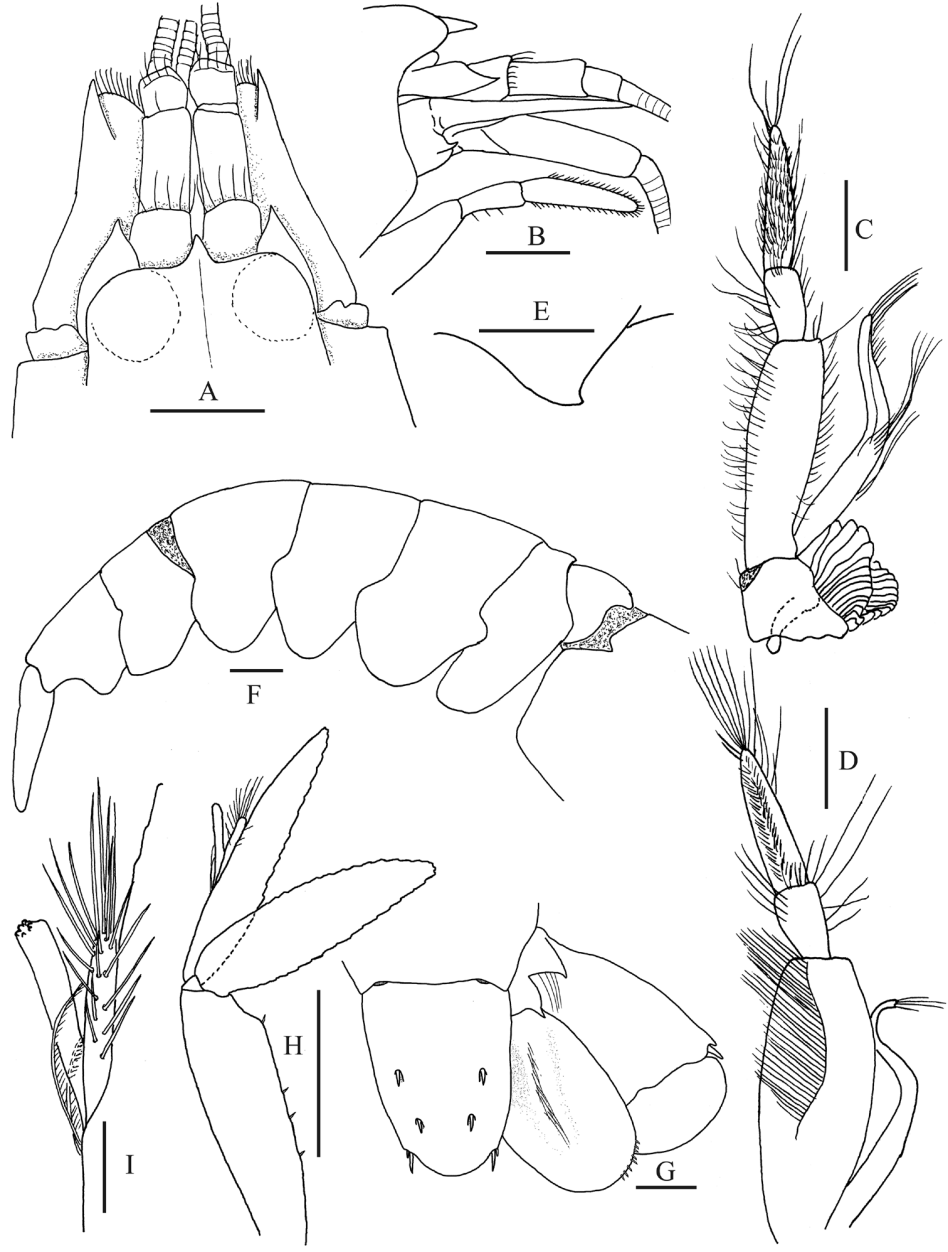
*Alpheus margaritae* sp. n. Paratype male, CL 6.5 mm (**D, H, I**), (CNCR 32595), Female paratype, CL 7.4 mm (**A–C, E–G**) (EMU-10581); **A** anterior portion of carapace and cephalic appendages, dorsal view **B** same in lateral view (setae omitted) **C, D** third maxilliped **E** antennular carina **F** abdomen, lateral view (setae omitted) **G** telson and uropods, dorsal view (setae partially omitted) **H** second pleopod **I** detail of appendices interna and masculina. Scale bars: **A–D, F–H** 1 mm; **E, I** 0.2 mm

**Figure 3.**
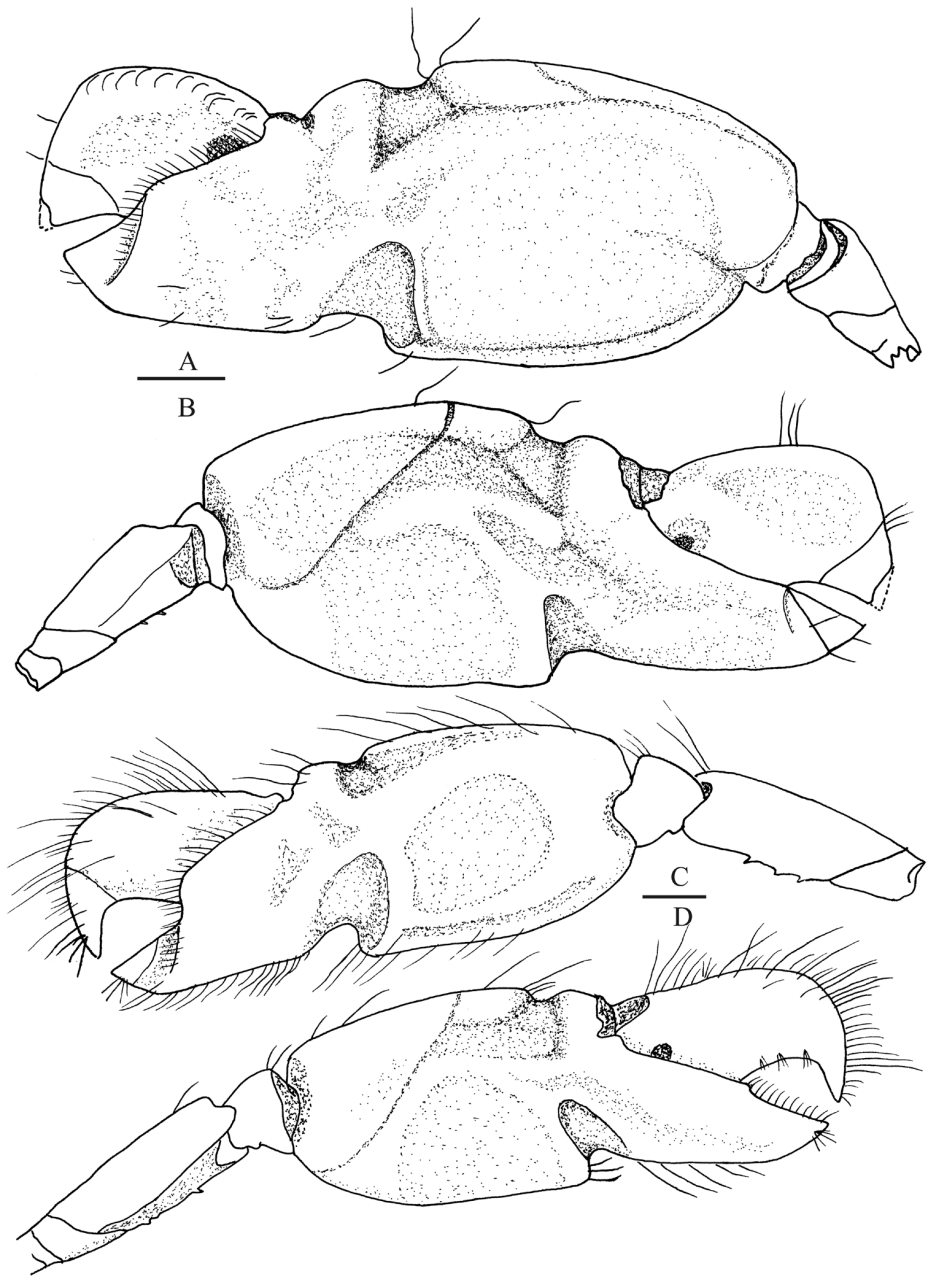
*Alpheus margaritae* sp. n. Female paratype, CL 7.4 mm (**A, B**) (EMU-10581); male paratype, CL 6.5 mm (**C, D**), (CNCR 32595); **A, C** major first pereopod, mesial view **B, D** same, lateral view. Scale bars 1 mm.

**Figure 4.**
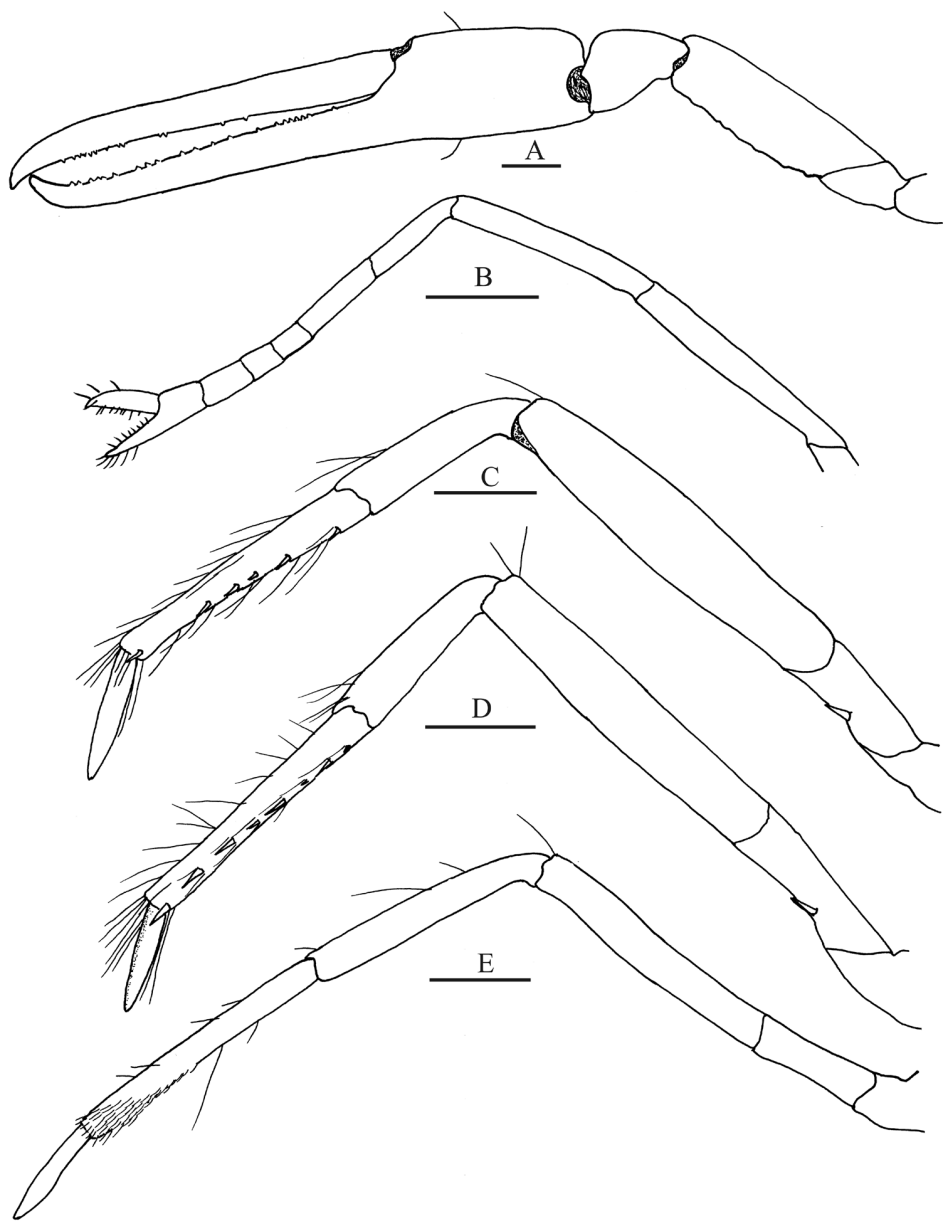
*Alpheus margaritae* sp. n. Female paratype, CL 7.4 mm, (EMU-10581); **A** minor first pereopod, mesial view **B** left second pereopod, lateral view **C** left third pereopod, lateral view **D** left fourth pereopod, lateral view **E** left fifth pereopod, lateral view. Scale bars 1 mm.

**Figure 5.**
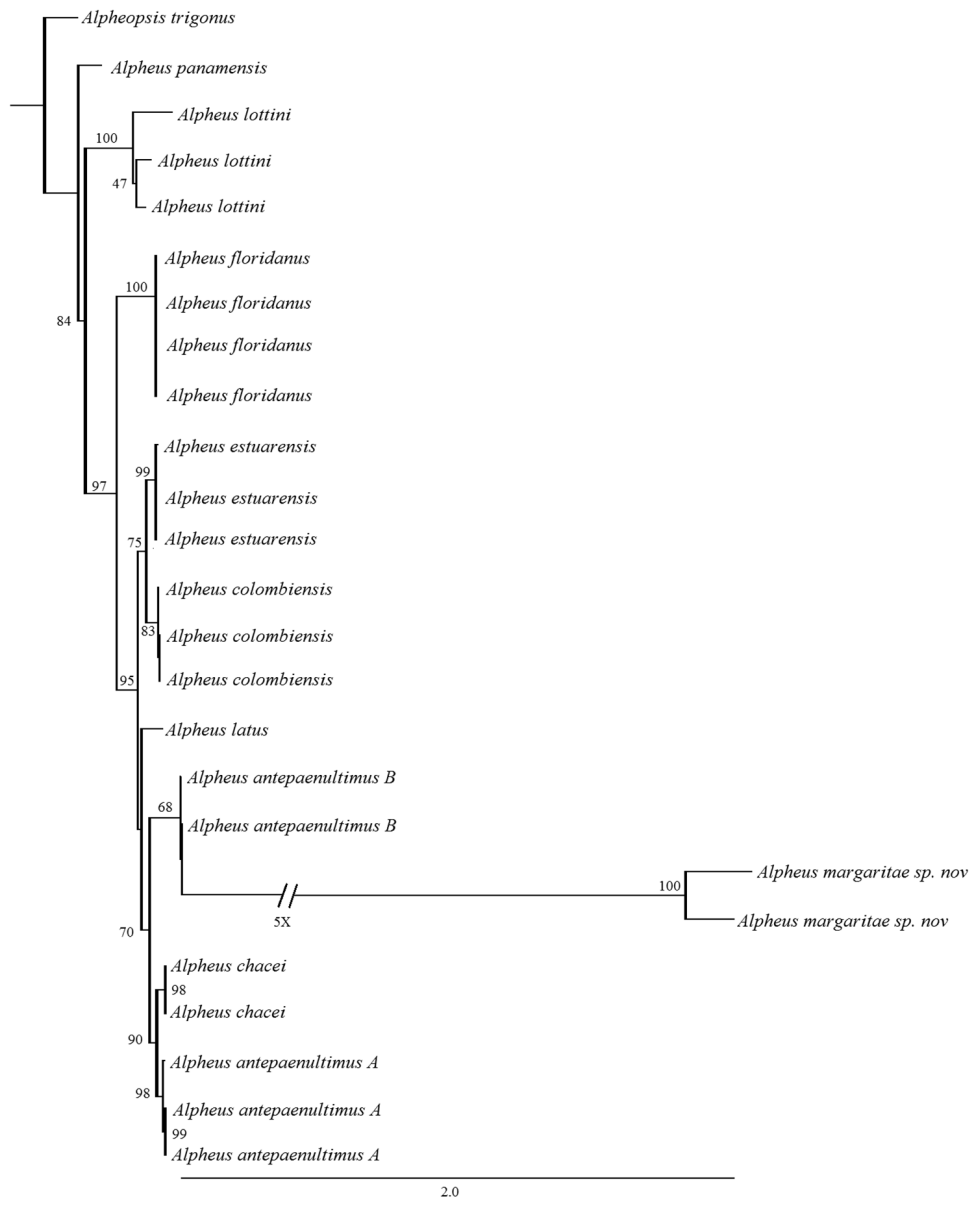
ML tree for *Alpheus* COI sequences. Numbers above each branch are MP bootstrap values. GeneBank accession numbers of *Alpheus chacei*, *A. antepaenultimus* A and *A. antepaenultimus* B as in Williams et al. (2001).

Figs 1 , 2 , 3 , 4 , 5

###### Material examined.

Holotype: Male (CL 4.6 mm), Costa Azul Island, Santa María-La Reforma, Sinaloa, Mexico, 25°5'56"N, 108°7'58"W, 0.1–0.3 m, mudflat with gravel at neap tide, March 30, 2015, (EMU- 10580). Paratypes: all same locality and data as holotype, 5 females (CL 3.0–5.7), 2 ovigerous females (CL 4.3–5.6 mm), 1 juvenile (CL 2.3 mm), (EMU-10581); paratypes, same locality and data as holotype, 1 male (CL 6.5 mm), 1 ovigerous female (CL 5.8 mm), (CNCR 32595).

###### Diagnosis.

Ocular hoods unarmed. Antepenultimate segment of third maxilliped broad. Scaphocerite with concave lateral margins, distolateral tooth overreaching the distal margin of the inner blade, inner blade almost reaching the distal end of antennular peduncle. Major cheliped markedly compressed, with grooves on both dorsal and ventral margins. Pereopods 3–5 with dactylus subspatulate; ischium of third and fourth pereopods with ventral spine.

###### Description.

*Carapace* glabrous (Fig. 2A, B), rostrum triangular, sharp, exceeding anterior margin of ocular hood, reaching 0.33 of visible portion of first antennular segment; rostral carina low and narrow, barely overpassing posterior end of eye; orbital hoods inflated dorsally and produced anteriorly, anterior margin convex, unarmed; orbitorostral groove shallow; pterygostomial margin slightly produced anteriorly below basis of basicerite. First antennular segment (Fig. 2A, B, E) bearing subtriangular carina on ventromesial margin, posterior margin of ventromesial carina convex, anterior margin concave, ventral margin with acute tip, directed anteriorly; second antennular segment approximately 2.0 times as long as wide, 1.5 times length of visible part of first segment, and 3.0 times as long as third segment; stylocerite almost reaching distal margin of first segment.

*Antenna* (Fig. 2A, B) with lateral margin of scaphocerite slightly concave; distolateral tooth reaching distal end of antennular peduncle; inner blade falling short of tip of distolateral tooth; cleft between inner blade and distolateral tooth arising from approximately 0.25 of scaphocerite; carpocerite overpassing distal end of antennular peduncle by 0.4 length of third antennular segment; basicerite with acute lateral tooth.

*Third maxilliped* (Fig. 2C, D) reaching distal margin of carpocerite; last segment tapering distally, mesial surface setose, almost twice as long as penultimate segment; penultimate segment approximately twice as long as broad; antepenultimate segment fairly enlarged and flattened, approximately 2.6 times as long as broad, longer than the sum of preceding two segments; lateral surface smooth, mesial surface with sinuous carina bearing long setae; exopod falling near to median part of penultimate segment in holotype and two female paratypes, shorter than penultimate segment in males and the rest of females; precoxa with one arthrobranch near distal end and small supplementary arthrobranch near proximal end.

*Major cheliped* of first pereopods (Fig. 3A–D) narrow, 2.4 times as long as broad and with trace of fine granules at anterior half; fingers clearly narrower than palm, occupying the distal 0.4 of chela (0.35 in juveniles); movable finger with superior margin slightly arched at proximal two thirds and then right angled, tip narrow and acute; pollex acute at tip, inferior margin almost straight along proximal two thirds and convex along distal third; palm with superior and inferior transverse grooves; superior transverse groove broad and low, continuing to shallow elongated triangular depression on mesial face and continuing to shallow broad rectangular depression on lateral face; mesial superior depression continuing to proximal portion of palm; lateral superior depression continuing to linea impressa; linea impressa continuing with mesial upper longitudinal depression; inferior transverse groove fairly deep and broad with proximal shoulder slightly projecting, connecting to inverse V-shaped inferior lateral palmar depression; lateral palmar face with shallow depression below superior palmar depression and behind inferior palmar depression, and with median depression between the above two depressions and continuing to below proximal portion of dactylus; mesial palmar surface with elongate depression near inferior margin; a slightly depressed area between upper and lower longitudinal depressions and lower areas between and in front of upper and inferior transverse grooves; ventromesial margin of merus with trace of fine granules, bearing two minute movable teeth at proximal half, unarmed distal end; ventral surface with trace of fine granules in largest specimens.

*Minor chela* of first pereopods (Fig. 4A) elongate, 5.6 times as long as broad, with trace of fine granules inferiorly; fingers occupying 0.7 of chela; cutting edges barely serrated, subparallel when closed, apex deflexed inward; movable finger longer than immovable finger in most specimens; tips of fingers blunt to acute. Palm lacking sculpturing, with trace of fine granules on mesial face, swollen laterally and thicker than fingers. Merus with ventromesial margin bearing short setae, with two small movable teeth at proximal half.

*Second pereopod* (Fig. 4B) reaching distal end of carpocerite beyond distal end of first segment of carpus. Chela shorter than the sum of three distal segments of carpus. Fingers of chela 1.4 times as long as palm. First segment of carpus 1.4 times as long as second; second segment 2.6 times as long as third; fourth segment almost as long as third; fifth segment 1.7 times as long as fourth.

Dactylus of *third pereopod* (Fig. 4C) subspatulate, 0.5 times as long as propodus; propodus 1.4 times as long as carpus, with inferior margin bearing six small spines along its extension. Merus 5.6 times as long as broad and 1.8 times as long as carpus; ischium with ventral spine.

*Fourth pereopod* (Fig. 4D) similar to third pereopod; ischium with ventral spine.

Ischium of fifth pereopod (Fig. 4E) unarmed; propodus setose distally, setae increasing in number distally; dactylus subspatulate.

*Pleura* of first to fourth abdominal somites rounded ventrally, not overlapping each other much on ventral regions, that of fifth somite subtriangular on posterior ventral margin (Fig. 2F). Abdominal sternite unarmed at midline. Appendix masculina (Fig. 2H, I) slightly shorter than appendix interna.

*Telson* (Fig. 2G) Approximately 1.5 times as long as broad at anterior end, armed with two pairs of dorsal spines and low longitudinal median depression on dorsal surface; posterior margin very convex, with two pairs of postero-lateral spines, inner spine stronger, twice as long as outer. Uropodal endopod with fairly distinct mesial depression at anterior half; uropodal exopod bearing movable spine outside of transverse suture, movable spine flanked laterally by short immovable tooth and internally by round lobe; suture forming almost two straight lobes.

###### Habitat.

Soft mud with gravel composed of shells and rocks, in intertidal.

###### Color in life.

Creamy-white with irregular sparse olive green to light brown patches on dorsal surface of carapace, abdomen and telson; uropodal endopodite and distal lobe of exopodite olive green to light brown, proximal lobe of exopodite creamy-white; patches on abdominal plates tend to be more dense towards the rear; first pereopod olive green. No clear differences were observed between sexes (Fig. 1).

###### Distribution.

Only known from Bahía Santa María-La Reforma coastal lagoon, Sinaloa, Mexico.

###### Etymology.

The species is named after Dr. Margarita Hermoso Salazar in recognition of her contributions to the knowledge of Mexican carideans.

###### Variations.

Body structures in *Alpheus margaritae* sp. n. are similar between sexes; sculpture in major cheliped becomes deeper in larger specimens. Disparities among the specimens are:

In nine of the twelve specimens the antepenultimate segment of the third maxilliped is approximately 2.5 times as long as broad, with a sinuous carina on the inner surface bearing long setae and the exopod not reaching the distal end of the antepenultimate segment (Fig. 2D), in three females the shape of that appendage is quite different; the antepenultimate segment of the third maxilliped is slender, without a well-defined inner carina and sparse long setae; the exopod is longer, reaching the middle of the penultimate segment (Fig. 2C); all specimens, with exception of the largest female, bear two spines on proximal half of inferior inner margin of merus of both major and minor chelae and no distal spines are present. In the largest female those proximal spines are absent, although there are some marks in the place where the spines should be located, that likely indicates that spines were lost; relative length of fingers in minor first pereopod increases with the size of the specimens; ranging from 64% in the smallest juvenile to 72% in one of the largest females, with exception of one female (CL = 5.0 mm) whose fingers occupied only 45% of minor chela length. The latter chela does not seem to be damaged and does not appear to represents a case of regeneration.

###### Remarks.

*Alpheus margaritae* sp. n. is morphologically similar to *A. antepaenultimus*, *A. mazatlanicus*, and *A. chacei*. These species share the absence of teeth on the ocular hoods, the antepenultimate segment of the third maxilliped being broad, the major cheliped markedly compressed with grooves on both dorsal and ventral margins, and pereopods 3–5 with subspatulate dactylus.

The new species and *A. antepaenultimus* have the inferior inner margin of merus with spines, while in *A. chacei* the merus lacks these spines. *Alpheus margaritae* sp. n. can be differentiated from *A. antepaenultimus* as in the former the scaphocerite has concave lateral margins, the distolateral tooth overreaches the distal margin of the inner blade, and the inner blade almost reaches the distal end of the antennular peduncle, whereas in *A. antepaenultimus* the lateral margins of the scaphocerite are almost straight, the distal spine almost reaches the distal margin of the inner blade, and the inner blade overpasses the distal end of antennular peduncle. In large specimens of the new species (CL > 5.0 mm) the superior transverse groove of the major cheliped is deeper and the sculpture is more conspicuous than in *A. antepaenultimus*. The proximodorsal margin of the movable finger of *A. margaritae* sp. n. is almost straight and the tip is narrow and acute, while in *A. antepaenultimus* the dorsal margin of the movable finger is arched along all its longitude and bluntly rounded at tip. Also, the number of spines on inferior inner margin of merus of major first pereopods in *A. antepaenultimus* is greater than in the new species (3 or 4 vs. 1 or 2).

*Alpheus margaritae* sp. n. can be differentiated from *A. mazatlanicus* as in the new species the second antennular segment is proportionally much shorter than wide (2 times vs. 4 times) and the lateral margin of the scaphocerite is slightly concave instead of straight; *A. margaritae* has a hook-like carina on the ventromesial face of the first article of antennular peduncle whereas in *A. mazatlanicus* this carina is broadly triangular; besides, *A. margaritae* has the inner margin of merus of the first pereopod with movable spines on proximal half while in *A. mazatlanicus* such spines are absent; and the ratio of the fingers in relation to the palm in the minor chela is shorter (60%) in *A. mazatlanicus* than in the new species (approximately 70%).

*Alpheus margaritae* is more related to *A. antepaenultimus* B. The latter is part of a species complex from the Eastern Pacific which include *A. floridanus* specimens from Eastern Pacific sensu Williams et al. 2001 and *A. hephaesthus* (Braken-Grissom et al. 2014). *A. antepaenultimus* A, *A. antepaenultimus* B, and *A. chacei* form a clade consistent with the morphological traits previously described. This clade could be the result of an ecological radiation, probably associated to the species flexibility to inhabit in tropical and subtropical conditions (Williams et al. 2001). The position of *A. margaritae* within the *A. antepaenultimus* complex suggests that this group of species, and probably *A. mazatlanicus*, could have been subject to such ecological radiation.

###### Phylogenetics relationships.

The COI matrix of *Alpheus* molecular data consisted of 668 characters. The ML phylogenetic hypothesis shows that *Alpheus margaritae* sp. n. is more related to *A. antepaenultimus* B with a bootstrap support of 63. On the other hand, *A. margaritae* sp. n. shows an important mutational distance that differentiates it from *A. antepaenultimus* B. This pair of species is grouped together in the clade formed by *A. chacei* and *A. antepaenultimus* (Fig. 5). COI matrix of *Alpheus* shows that *A. margaritae* sp. n. belongs to the species complex formed by *A. antepaenultimus* A, B, and *A. chacei*. This complex corresponds to the mangrove group (type a) of the clade I proposed by Williams et al. (2001). The *A. antepaenultimus* complex is characterized by the combination of: 1) the absence of spines on the occular hoods, 2) the antepenultimate segment of the third maxilliped is broadened, 3) major cheliped compressed, with transverse groove on superior and inferior margins proximal to fingers, 4) immovable finger of minor chela never balaeniceps, and fingers occupying more than 0.6 of chela, 5) pereopod 3-5 with subspatulate dactylus, 6) ischium of pereopods 3-4 with spine on the ischium, 7) uropodal spines not colored.

#### Genus *Leptalpheus* Williams, 1965

##### Leptalpheus melendezensis sp. n.

Taxon classificationAnimaliaDecapodaAlpheidae

http://zoobank.org/2DE36886-5588-43C2-B031-10FD45C1EEBA

Figs 6 , 7 , 8 , 9

###### Material examined.

Holotype: Male (CL 4.1 mm), Meléndez Island, Santa María-La Reforma, Sinaloa, Mexico, 24°48'07"N, 108°03'22.3"W, sand, 0.2 m at low tide, January 18, 2015, (EMU-10582). Paratype: 1 male (CL 2.9 mm), same data as holotype, (EMU-10583).

###### Diagnosis.

Frontal margin of carapace broadly rounded, weakly produced, without dorsal crests. Antenna with carpocerite longer than scaphocerite, slightly shorter than antennular peduncle. Major cheliped slender; ischium armed with strong ventromesial spine directed upward; fingers slightly twisted laterally, not gaping when closed; without adhesive discs; dactylus with strong proximal tooth on cutting edge, tip acute, crossing distally with tip of pollex; propodus of pereopods 3 and 4 with two ventral spines; propodus of fifth pereopod with two distal rows of setae on ventral margin.

###### Description.

**Figure 6.**
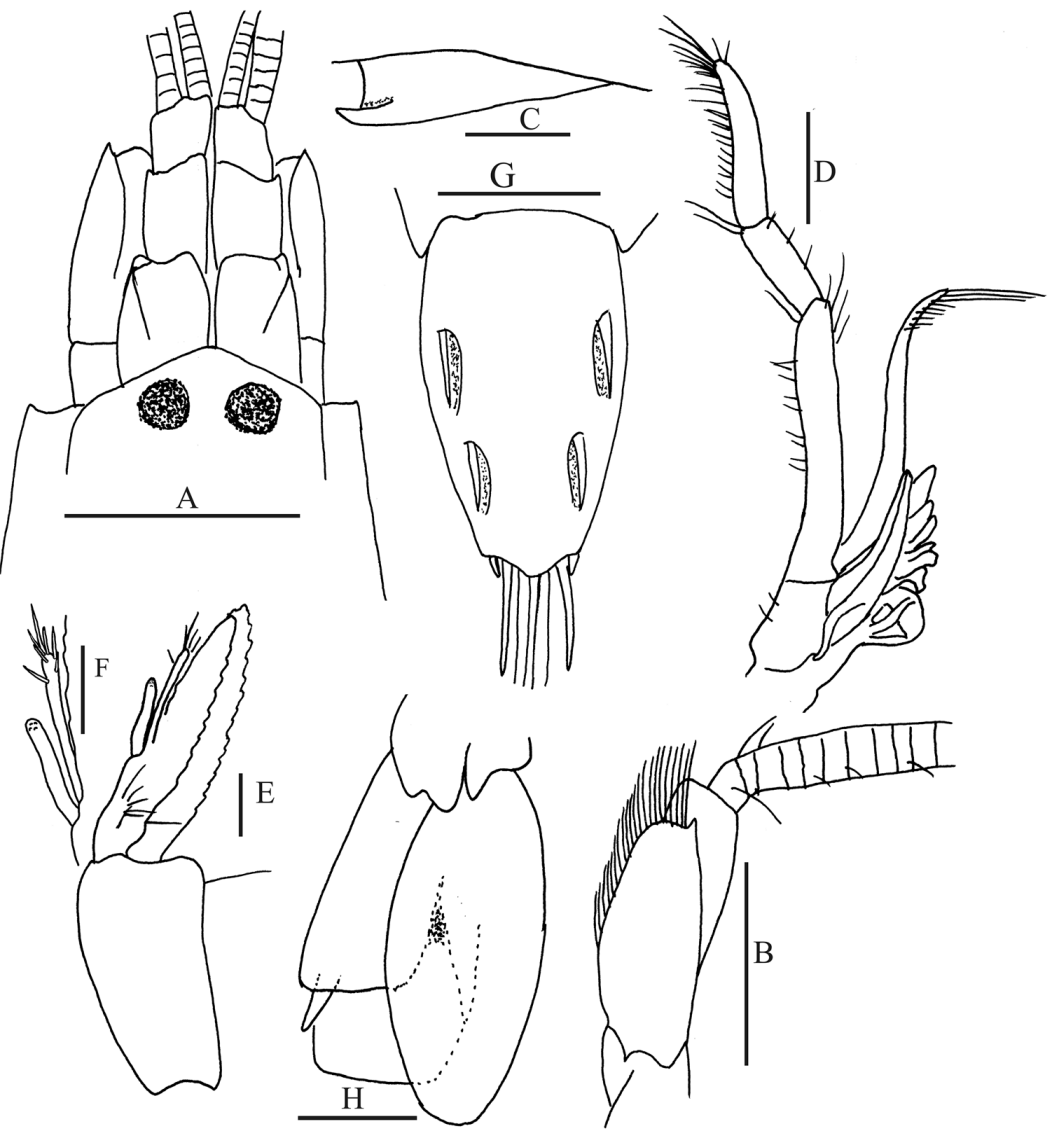
*Leptalpheus melendezensis* sp. n. Holotype male, CL 4.1 mm (EMU10582); **A** anterior portion of carapace and cephalic appendages, dorsal view **B** right antenna, lateral view **C** tooth on ventromesial carina of first segment of antennular peduncle, mesial view **D** third maxilliped **E** second pleopod **F** detail of masculina and internal appendix **G** telson, dorsal view **H** left uropod, dorsal view. Scale bars **A, B** 1 mm; **C, E** 0.2 mm; **D, G, H** 0.5 mm.

Frontal margin of *carapace* (Fig. 6A) broadly rounded, obtuse, weakly produced, carapace smooth, without dorsal crests or carina; eyestalks with anteromesial margin rounded.

*Antennular peduncles* (Fig. 6A) moderately stout, flattened dorsoventrally; second article longer than broad; stylocerite appressed, not reaching distal margin of first article of the antennular peduncle; ventromesial carina (Fig. 6C) terminating in an upward curving tooth projected beyond the carina; lateral flagellum with two articles.

*Antenna* (Fig. 6B) with stout basicerite bearing strong distoventral tooth. Scaphocerite (Fig. 6A, B) ovate, with acute distolateral tooth reaching beyond the anterior margin of blade, blade with mesial margin curved; carpocerite longer than scaphocerite, slightly shorter than antennular peduncle.

Mouthparts not dissected, typical for genus in external view. Third maxilliped (Fig. 6D) with lateral plate on coxa produced upward, not reaching the distal margin of the branchiae; ultimate article with rows of brush-like setae increasing in size distally; exopod longer than first article.

**Figure 7.**
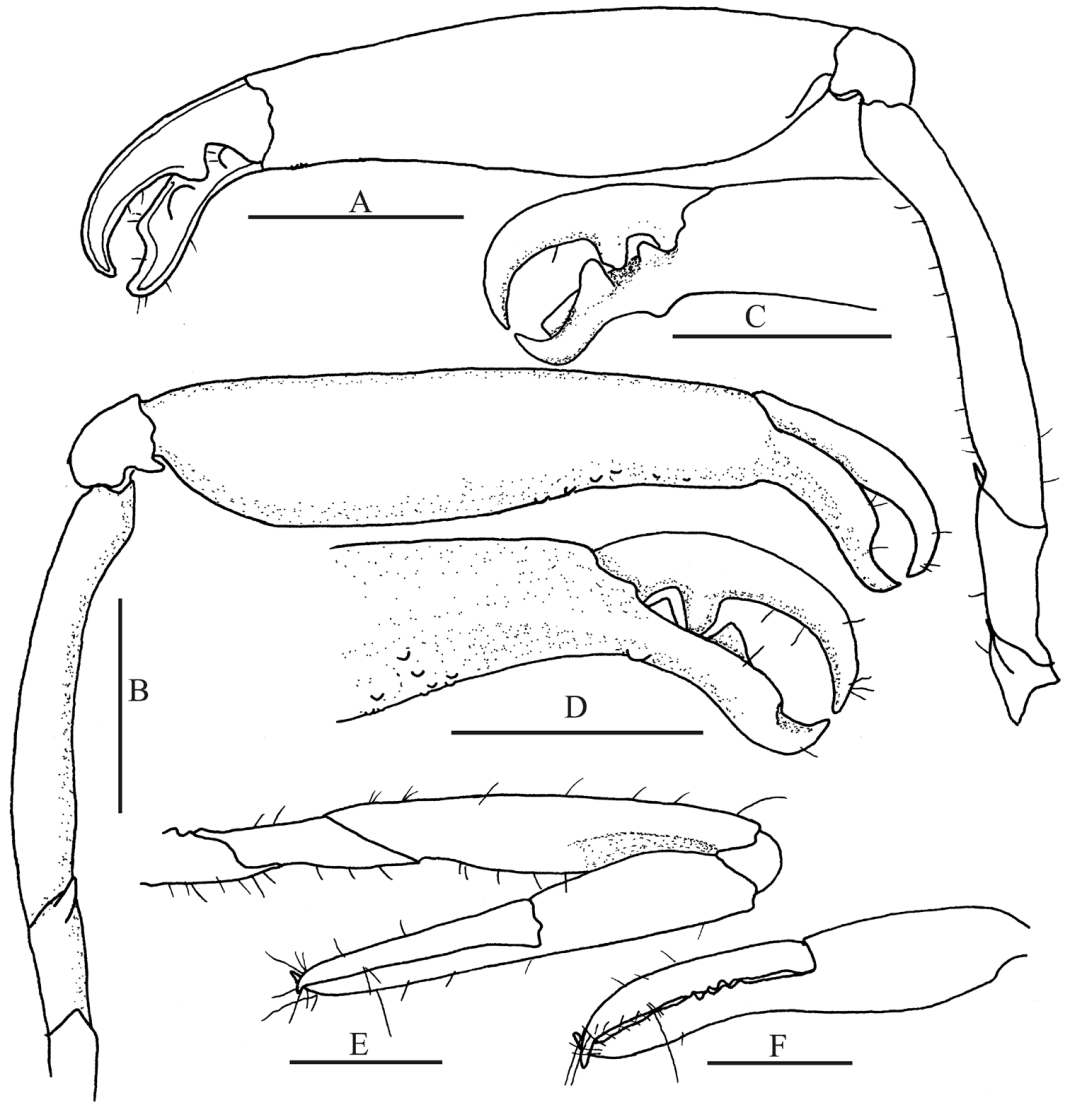
*Leptalpheus melendezensis* sp. n. Holotype male, CL 4.1 mm (EMU-10582); **A** major first pereopod, outer view **B** same, inner view **C** detail of distal portion of same cheliped, outer view **D** same, inner view **E** minor first pereopod, outer view **F** detail of same cheliped, outer view. Scale bars **A, B** 1 mm; **C–F** 0.5 mm.

*Major cheliped* (Fig. 7A–D) slender; ischium armed with strong ventromesial spine directed upward; merus slender, with concave depression along ventral margin, ventral margin minutely granulated; carpus short, cup-shaped, dorsally convex, with two distoventral processes, the external blunt and the mesial tooth-like; chela robust, longer than merus; palm 2.5 times as long as fingers, ventrally depressed, narrowing distally, ventral margin with sparse tubercles, dorsal margin smooth; without adhesive discs; fingers slightly twisted laterally, not gaping when closed; pollex with proximal half ventrally deflexed, outer cutting edge (Fig. 7C) armed with two teeth, distal stronger, mesial margin with proximoventral low tubercle and a shallowly excavated projection along proximal two thirds, tip acute, directed upward; dactylus noticeably curved, dorsum smooth, cutting edge with strong proximal tooth, tip acute, crossing distally with tip of pollex.

*Minor cheliped* (Fig. 7E, F) with segments unarmed; merus almost as long as chela; carpus short; fingers pointing, slightly longer than palm, median portion of cutting edges armed with 4–5 small, irregularly spaced teeth, tips crossing distally.

**Figure 8.**
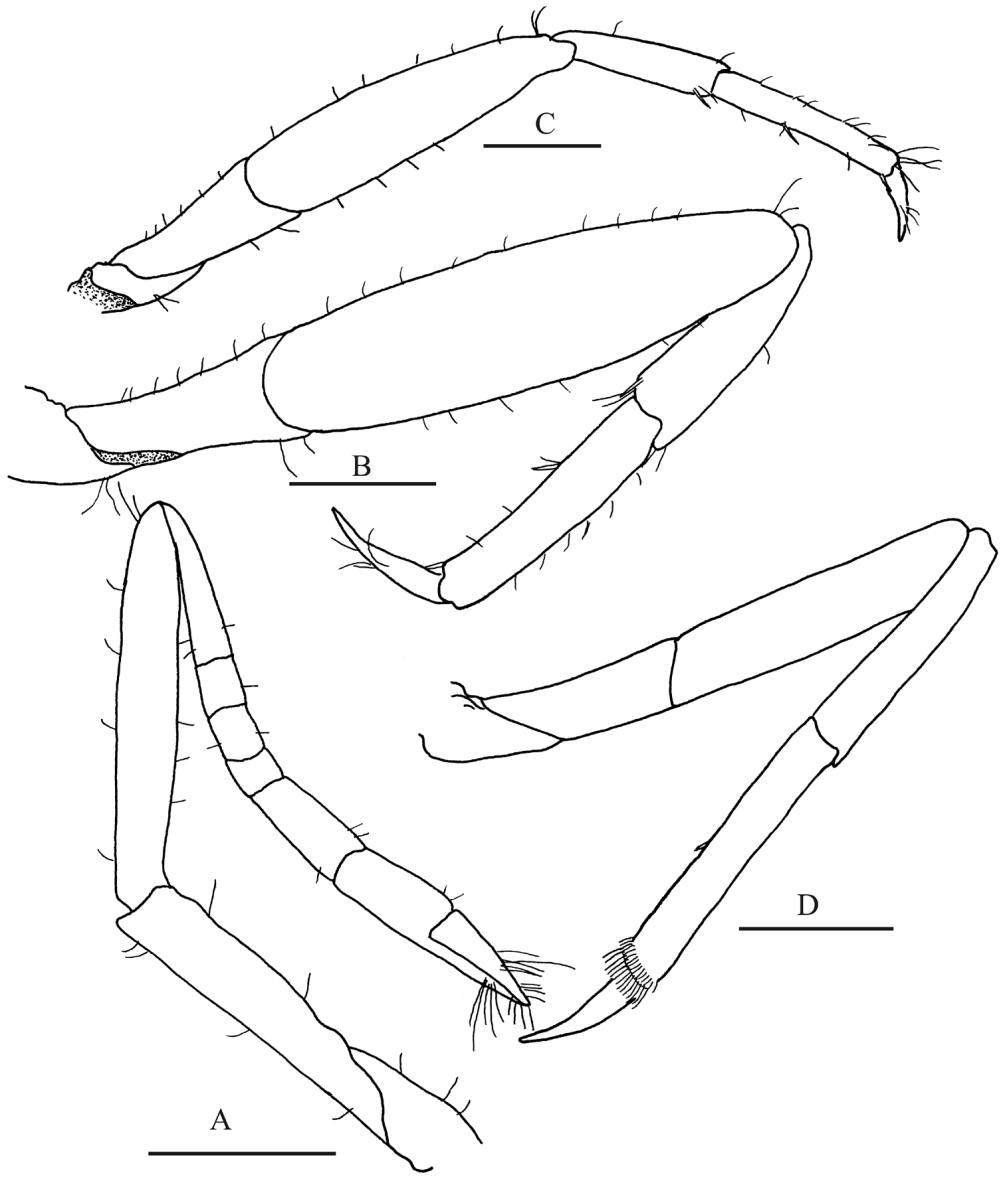
*Leptalpheus melendezensis* sp. n. Holotype male, CL 4.1 mm (EMU-10582); **A** second pereopod, lateral view **B** third pereopod, lateral view **C** fourth pereopod, lateral view **D** fifth pereopod, lateral view. Scale bar 0.5 mm.

*Second pereopod* (Fig. 8A) unarmed; ischium 0.75 as long as merus; merus shorter than carpus; carpus 5-articulated, articles ratio from proximal to distal approximately 6:2:2:1:4; distal portion of chela with long setae.

*Third and fourth pereopods* (Fig. 8B, C) similar, compressed; third pereopod slightly longer and stouter than fourth, merus slightly longer than combined length of carpus and propodus, ventrodistal margin of carpus with ventrodistal spine, propodus with 2 spines on median and distal ventral margin and sparse long setae along margins; dactylus curve, slender, acute distally, nearly half as long as propodus.

*Fifth pereopod* (Fig. 8D) slender, not compressed, ventral margin of propodus with 1 median spiniform seta and distal margin with two rows of setae, distalmost larger; dactylus acute, curved.

*Male second pleopod* (Fig. 6E) with slender appendix masculina, with two subterminal and four terminal setae; appendix interna 0.75 length of appendix masculina (Fig. 6F).

*Telson* (Fig. 6G) widest in proximal third, slightly tapering distally, dorsal surface with two pairs of strong spines inserted in deep pits close to lateral margins, posterior margin rounded, with two pairs of spiniform setae at posterolateral angles, lateral one much shorter than mesial one, posterior margin with 4 plumose setae.

*Uropod* (Fig. 6H) with lateral lobe of protopod ending in two small lobes; exopod with posterodistal margin straight, deeply incised, with a large spine near mesial margin.

###### Habitat.

Sandy beach, associated with burrows of *N. tabogensis*.

###### Distribution.

Known only from Meléndez Island, Bahía Santa María-La Reforma, Sinaloa, Mexico.

###### Etymology.

The name of the species is derived from Meléndez Island, the type locality.

###### Remarks.

*Leptalpheus melendezensis* sp. n. seemed to be related to *L. mexicanus* because the general plan of major cheliped of both species is similar; they both have a curved, slender merus, short carpus and ventrally depressed manus, narrowing distally, with sparse ventral tubercles and convex dactylus with a strong proximal tooth. However, a detailed analysis reveals differences between the species. The ischium of the major cheliped in *L. melendezensis* sp. n. bears a noticeable ventromesial spine, while in *L. mexicanus* it is absent; pollex and dactylus of the major cheliped do not gape in the new species but form a wide open gape in *L. mexicanus*; the pollex of the major cheliped in *L. mexicanus* is ventrally convex and spoon-shaped at distal end, whereas in the new species it is ventrally concave, with a lateral projection along proximal two thirds and acute at its distal end. Other differences are that the carapace of *L. mexicanus* has an acute, carinate triangular rostrum and two small orbital crests above the eyes, whereas in *L. melendezensis* sp. n. the carapace has a blunt, scarcely projected rostrum without a median carina and without supraocular crests; the tooth on the antennular ventromesial carina is more anteriorly projected in the new species; the propodus of pereopods 3 and 4 of *L. melendezensis* sp. n. bears only two ventral spines as opposed to three in *L. mexicanus*; and the fifth pereopod has two distal rows of setae instead of four as in *L. mexicanus*.

*Leptalpheus melendezensis* sp. n. is the eighth species assigned to the genus in the eastern Pacific and the fourth recorded from the Pacific coasts of Mexico, and is the only eastern Pacific species of *Leptalpheus* that presents a major cheliped without adhesive disks and isquium armed with a ventromesial spine.

###### Phylogenetic relationships.

**Figure 9.**
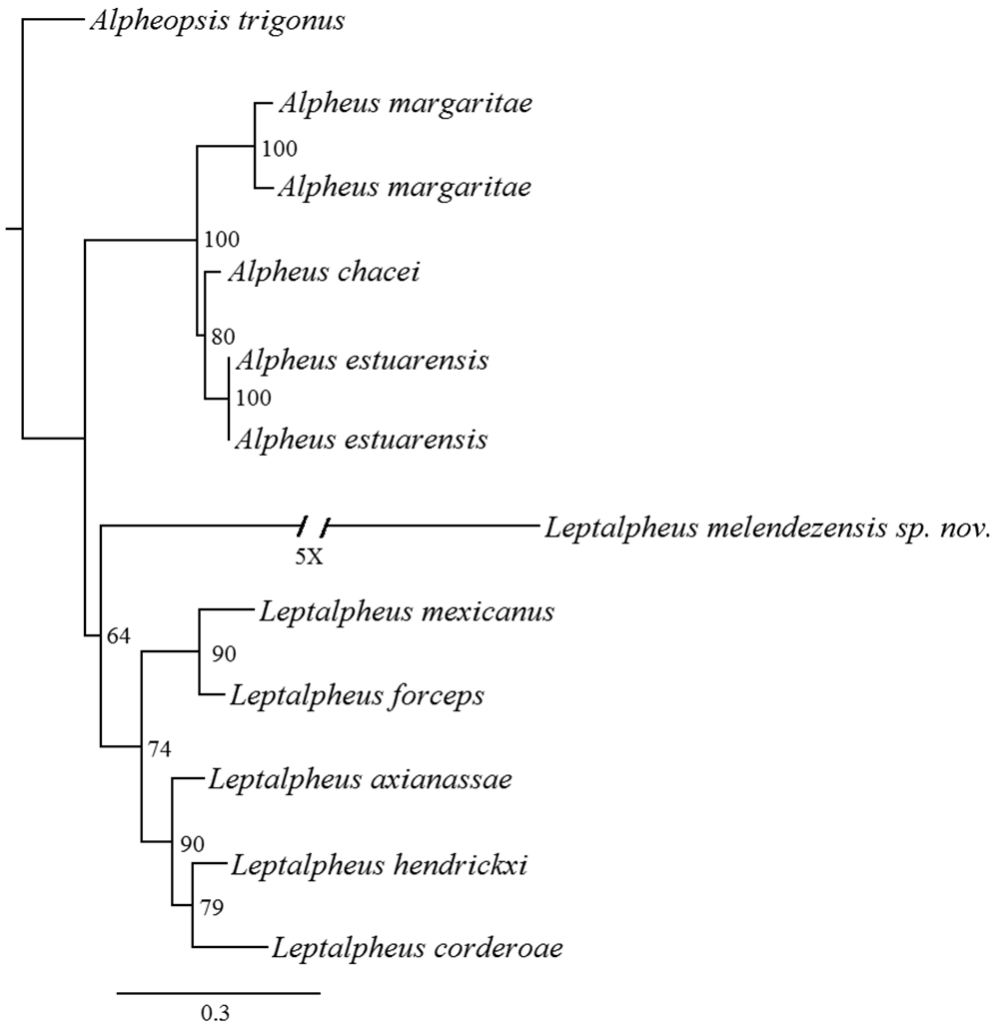
ML tree for *Leptalpheus* 16S sequences. Numbers in the nodes are 1000 bootstrap iterations result values.

The 16S matrix of *Leptalpheus* molecular data consisted of 688 characters. This relationship had a bootstrap support of 64 (Fig. 9). Also an insertion of five base pairs (TATTT) was identified which was not found in other *Leptalpheus* sequences.

The phylogenetic analysis presented here is a partial contribution to establish a phylogeny of the genus due to the almost total absence of genetic information from other species described in previous works, however this approach and morphological analysis provide sufficient information to recognize that *L. melendezensis* represent a species new to science.

##### Leptalpheus cf. mexicanus

Taxon classificationAnimaliaDecapodaAlpheidae

Ríos and Carvacho, 1983

###### Material examined.

Two males (CL 3.4 and 3.7 mm) and 4 ovigerous females (CL 2.9–3.6 mm), SE Talchichilte Island, Bahía Santa María-La Reforma, Sinaloa, Mexico, 24°50'5.71"N, 108°03'4.23"W, muddy sand, 0.2 m at low tide, October 5, 2014, (EMU-10584).

###### Habitat.

Previous work and this study agree that this species appears to be associated with mangrove forest (Ríos and Carvacho 1983; Salgado-Barragán et al. 2014). In Santa María-La Reforma, the species was collected in muddy sand, associated with burrows of *N. tabogensis*, near mangrove forest

###### Distribution.

Estero Mulegé, Baja California Sur (type locality), Bahía Santa María-La Reforma and Mazatlán, Sinaloa, and Manzanillo, Colima, Mexico, to Bahía Málaga, Colombia (Salgado-Barragán et al. 2014; present study).

###### Remarks.

We could only observe very small crests on carapace of the two males, which were absent in all females. In our opinion, the presence of these structures could be related to the size of the specimens. Specimens of L. cf. mexicanus and *L. melendezensis* sp. n. were collected in *N. tabogensis* burrows, but L. cf. mexicanus was found in muddy sand substrata, whereas the new species was found in a beach of fine sand.

### Family Processidae Ortmann, 1896

#### Genus *Ambidexter* Manning & Chace, 1971

##### Ambidexter panamensis

Taxon classificationAnimaliaDecapodaAlpheidae

Abele, 1972

###### Material examined.

1 female (CL 3.7 mm), 1 ovigerous female (CL 4.2 mm), Garrapata Island, 25°9'13"N, 108°15'25"W, silty sand, 0–0.2 m at low tide, June 25, 2013, (EMU-10585A); 2 females (CL 3.9 mm) and 2 ovigerous females (CL 5.2–5.3 mm), same locality, January 29, 2014, (EMU-10585B); 2 females (CL 3.5–3.6 mm) and 1 ovigerous female (CL 3.9 mm), same locality, March 30, 2015, (EMU-10585C); 1 male (CL 4.5 mm), Talchichilte Island, Sta. 1, 25°1'15"N, 108°7'6"W, silty sand, 0.1–0.3 m at low tide, January 18, 2015, (EMU-10585D); 1 female (CL 3.2 mm) and 2 ovigerous females (CL 3.5–3.9 mm), Sta. 2, (24°56'36"N, 108°2'54"W), mud-sand, 0.1–0.3 m at low tide, October 05, 2014, (EMU-10585E); 1 male (CL 3.3 mm), same locality, January 18, 2015, (EMU-10585F); 1 male (CL 3.6 mm) and 1 ovigerous female (CL 4.8 mm), 24°44'46"N, 107°59'41"W, mud-sand with gravel, 0.1–0.3 m at neap tide, January 18, 2015, (EMU-10585G); 1 female (CL 2.7 mm) and 1 ovigerous female (CL 4.2 mm), Saliaca Island, 25°8'55"N, 108°16'13"W, sand, 0.1–0.3 m at neap tide, March 30, 2015, (EMU-10585H); 4 ovigerous females (CL 4.7–5.6 mm), Meléndez Island 24°48'6"N, 108°3'21"W, sand, 0.1–0.3 m at neap tide, January 18, 2015, (EMU-10585I); 1 female (CL 3.4 mm) and 2 ovigerous females (CL 4.8–5.1 mm), Costa Azul Island, 25°5'56"N, 108°7'58"W, mud with gravel, 0.1–0.3 m at neap tide, March 30, 2015, (EMU-10585J).

###### Distribution.

Recorded from San Diego, California, and Gulf of California to Naos Island, Panama and Galapagos Islands (Wicksten and Hendrickx 2003)

###### Remarks.

According to Abele (1972), specimens of *A. panamensis* commonly inhabit sandy-muddy substrates with burrows of larger invertebrates. In the type locality, one specimen was found in the tube of a polychaete worm, but other crustaceans, such as callianasids, brachyurans and stomatopods were present in the same site. During our samplings, several specimens of callianassids were also collected together with, or near sites where *A. panamensis* was found. This is the first record of the species at Bahía Santa María-La Reforma, Sinaloa.

##### Ambidexter swifti

Taxon classificationAnimaliaDecapodaAlpheidae

Abele, 1972

###### Material examined.

One female (CL 3.9 mm), Meléndez Island, 24°48'6"N, 108°3'21"W, sand, 0.1–0.3 m at neap tide, January 18, 2015: (EMU-10586).

###### Distribution.

Recorded from San Benito Island, Baja California and the Gulf of California including Bahía Santa María-La Reforma to Paitilla, Panama and Galapagos Islands (Wicksten and Hendrickx 2003; this study). This species has been found intertidally in sandy substrate (Abele 1972; Wicksten 1991; this study).

###### Remarks.

This is the second record of the species from the Gulf of California, and the first record of a specimen found inhabiting together with a callianasid shrimp (*N. tabogensis*).

## Supplementary Material

XML Treatment for Alpheus margaritae

XML Treatment for Leptalpheus melendezensis

XML Treatment for Leptalpheus cf. mexicanus

XML Treatment for Ambidexter panamensis

XML Treatment for Ambidexter swifti
